# Regeneration of Articular Cartilage by Human ESC‐Derived Mesenchymal Progenitors Treated Sequentially with BMP‐2 and Wnt5a

**DOI:** 10.5966/sctm.2016-0020

**Published:** 2016-08-05

**Authors:** Jason D. Gibson, Michael B. O'Sullivan, Farhang Alaee, David N. Paglia, Ryu Yoshida, Rosa M. Guzzo, Hicham Drissi

**Affiliations:** ^1^ Department of Orthopaedic Surgery, UConn Musculoskeletal Institute, UConn Stem Cell Institute, UConn Health, University of Connecticut, Farmington, Connecticut, USA

**Keywords:** Wnt5a, BMP‐2, Articular cartilage, Embryonic stem cells, Mesenchymal stem cells

## Abstract

The success of cell‐based therapies to restore joint cartilage requires an optimal source of reparative progenitor cells and tight control of their differentiation into a permanent cartilage phenotype. Bone morphogenetic protein 2 (BMP‐2) has been extensively shown to promote mesenchymal cell differentiation into chondrocytes in vitro and in vivo. Conversely, developmental studies have demonstrated decreased chondrocyte maturation by Wingless‐Type MMTV Integration Site Family, Member 5A (Wnt5a). Thus, we hypothesized that treatment of human embryonic stem cell (hESC)‐derived chondroprogenitors with BMP‐2 followed by Wnt5a may control the maturational progression of these cells into a hyaline‐like chondrocyte phenotype. We examined the effects of sustained exposure of hESC‐derived mesenchymal‐like progenitors to recombinant Wnt5a or BMP‐2 in vitro. Our data indicate that BMP‐2 promoted a strong chondrogenic response leading to terminal maturation, whereas recombinant Wnt5a induced a mild chondrogenic response without promoting hypertrophy. Moreover, Wnt5a suppressed BMP‐2‐mediated chondrocyte maturation, preventing the formation of fibrocartilaginous tissue in high‐density cultures treated sequentially with BMP‐2 and Wnt5a. Implantation of scaffoldless pellets of hESC‐derived chondroprogenitors pretreated with BMP‐2 followed by Wnt5a into rat chondral defects induced an articular‐like phenotype in vivo. Together, the data establish a novel role for Wnt5a in controlling the progression from multipotency into an articular‐like cartilage phenotype in vitro and in vivo. Stem Cells Translational Medicine *2017;6:40–50*

**Significance Statement**

Posttraumatic osteoarthritis poses a significant clinical dilemma. Despite considerable advances, cell‐based reparative techniques for articular cartilaginous injuries often fail to regenerate permanent cartilage. With sequential treatment of H9 mesenchymal stem cell progenitor cells with BMP‐2 and Wnt5a, terminal maturation of chondrocytes was inhibited. Moreover, articular cartilage was regenerated by using a scaffoldless cell implantation technique in vivo. This study represents a significant advance in the quest to regenerate permanent articular cartilage.

## Introduction

Given the limited capacity of joint cartilage to self‐regenerate 1, 2, the quest for an effective cell‐based strategy to regenerate articular cartilage for the prevention of posttraumatic osteoarthritis has proven to be highly challenging. Some of the major considerations for effective cell‐based therapies include: (a) the availability of chondroprogenitor cells, and (b) the appropriate signals to control progenitor cell commitment and differentiation into articular‐like chondrocytes. Autologous chondrocyte implantation (ACI) is a widely used procedure for the repair of large cartilage defects 3 4 5 6 7. Despite therapeutic benefits of the procedure, there are significant limitations, including the inability of the transplanted chondrocytes to maintain phenotypic stability and to provide tissue integrity. Implantation of autologous cells often leads to the formation of mechanically unstable fibrocartilage 7 8 9. Additionally, the implanted cells exhibit a propensity to differentiate into terminally mature hypertrophic chondrocytes, thus preventing permanent cartilage tissue repair 7, 10 11 12 13 14 15. Finally, the limited proliferative capacity of autologous chondrocytes, which decreases with age, further justifies the demand for alternative cell sources to treat large cartilage defects 16 17 18. Therefore, there is a need to develop new and effective strategies to regenerate articular cartilage using progenitor cells with the capacity to differentiate into permanent cartilage matrix‐producing cells.

Given the limitations of autologous chondrocyte implantation, several groups have sought to use embryonic stem cell‐based approaches to regenerate cartilage. The differentiation of human embryonic stem cells (hESCs) into chondrocyte‐like cells has been accomplished through several methods 19 20 21 22 23 24 25 26 27, but evaluation of these techniques in the setting of in vivo articular cartilage repair has been relatively unexplored. The use of growth factors to promote chondrogenesis, or scaffolds to partition the deposition of tissue formation, have been studied in translational rodent models where hESC‐derived cells were implanted in surgically‐induced osteochondral defects. However, the implanted cells exhibited pronounced hypertrophic features, as evidenced by histologic evaluation 28 29 30. Pilichi et al. 31 implanted undifferentiated sheep‐derived embryonic stem cells (ESCs) from monolayer culture directly into an osteochondral defect, and observed regeneration of cartilage within the defect region. This cartilage exhibited some features of hypertrophy and did not optimally integrate with the adjacent host tissue.

High‐density culture systems, combined with growth factor‐based treatment for the induction of chondrogenesis, have been extensively used to study members of the transforming growth factor β (TGF‐β) superfamily 21, 22, 25, 26, 32 33 34 35. Although bone morphogenetic protein 2 (BMP‐2) has been widely shown to promote the chondrogenic commitment of hESC, this was concomitant with a potent induction of terminal maturation and subsequent production of matrix proteases 21, 22, 25, 26, 32, 35. The use of TGF‐β3, in combination with platelet‐derived growth factor BB, followed by BMP‐4 has been successfully used as a strategy to limit expression of the type I collagen, which is a fibrocartilaginous marker. However, this treatment failed to inhibit terminal maturation, thereby limiting its clinical applicability 34. More recently, parathyroid hormone‐related peptide (PTHrP) was used as a TGF‐β alternative to inhibit terminal hypertrophy. However, PTHrP also directly inhibited chondrogenic induction of mesenchymal stem cells (MSCs), limiting its use in regenerative cartilage applications 36. Collectively, although complex combinations of factors provided at specific stages of differentiation may ultimately be used to promote chondrogenesis, the differentiation of stem cells into articular‐like chondrocytes remains a significant challenge for the regeneration of permanent cartilage. For this reason, identifying a factor that can specifically inhibit hypertrophic differentiation without impairing chondrogenesis is important.

Noncanonical Wnt5a is a particularly attractive growth factor to mediate the inhibition of chondrocyte hypertrophy, while maintaining chondrogenesis in chondroprogenitor‐like cells. Mice lacking *Wnt5a* displayed delayed chondrocyte differentiation and abrogated chondrocyte hypertrophy during embryonic development 37. Furthermore, *Wnt5a* gain‐of‐function in type II collagen‐expressing chondrocytes resulted in decreased ossification, accompanied by increased articular cartilage thickness and a reduction in chondrocyte hypertrophy 38. Moreover, Wnt5a was able to induce chondrogenesis in limb bud progenitor cells, while inhibiting their terminal maturation 39, 40. Based on these data, we postulated that Wnt5a may act in a stage‐dependent manner to control chondrocyte differentiation in multipotent mesenchymal progenitors derived from human ESCs. In the present study, we examined whether the sequential treatment of hESC‐derived mesenchymal‐like progenitors with BMP‐2, followed by Wnt5a, constitutes an effective strategy to promote differentiation into articular‐like chondrocytes in vitro and to mediate hyaline cartilage regeneration in a translational model of cartilage repair in rats 41.

## Materials and Methods

### Derivation and Expansion of MSC Progenitor Cells From H9 hESCs

H9 (NIH 0062) human embryonic stem cells were maintained on irradiated mouse embryonic fibroblasts in hESC medium 42. H9 hESC colonies were dissociated by using Accumax (EMD Millipore, Billerica, MA, http://www.emdmillipore.com) and plated at 1 × 10^4^ cells per cm^2^ in MSC derivation medium consisting of high‐glucose Dulbecco's modified Eagle's medium (DMEM‐HG; Thermo Fisher Scientific Life Sciences, Oakwood Village, OH, https://www.thermofisher.com) supplemented with 10% defined fetal bovine serum (FBS; GE Life Sciences, Pasching, Austria, http://www.gelifesciences.com), 1% nonessential amino acids, 1% penicillin‐streptomycin, and 5 ng/ml human recombinant basic fibroblast growth factor (bFGF) as previously described 43, 44. With subsequent passages, the adherent populations of cells acquired a homogenous MSC‐like morphology. The H9‐derived MSC‐like cells (H9‐MSC) were passaged weekly, and medium was exchanged every 2–3 days.

### Flow Cytometry

H9‐derived MSCs and human bone marrow‐derived MSCs (Lonza, Walkersville, MD, http://www.lonza.com) were grown to confluence, harvested by using 0.25% trypsin/EDTA, and resuspended in buffer containing phosphate‐buffered saline (PBS), 2% HEPES buffer, 2% FBS, and 0.1% bovine serum albumin (BSA) as previously described 43. Cells (1 × 10^6^) were incubated with phycoerythrin (PE) mouse anti‐human CD90, PE mouse anti‐human CD73, fluorescein isothiocyanate (FITC) mouse anti‐human CD44, FITC mouse anti‐human CD45, FITC mouse anti‐human HLA‐ABC, PE mouse anti‐human CD29, PE mouse anti‐human CD166, PE mouse anti‐human HLA‐DR, FITC mouse anti‐human CD105, or FITC mouse anti‐human CD31 (BD Biosciences, San Jose, CA, http://www.bdbiosciences.com). Nonspecific fluorescence was determined by using isotype‐matched monoclonal antibodies. A total of 10,000 events were collected on a BD fluorescence‐activated cell sorting Calibur Flow Cytometer instrument by using CellQuest software (BD Biosciences). Analyses of results and corresponding graphs were generated by using FlowJo software (Tree Star, Ashland, OR, http://www.flowjo.com) 43 44 45.

### Osteogenic and Adiopogenic Multipotential Differentiation Assays

Osteogenesis was induced in monolayer H9‐MSC cultures (120,000 cells per cm^2^) in DMEM containing 10% FBS (GE Life Sciences), 1 mM sodium pyruvate, 10^−7^ M dexamethasone, 50 μg/ml ascorbic acid 2‐phosphate, 10 mM β‐glycerophosphate, and 1% penicillin/streptomycin as previously described 43. At 21 days, cultures were fixed and stained in alkaline phosphatase solution (Sigma‐Aldrich, St. Louis, MO, http://www.sigmaaldrich.com) as an indicator of osteoblast differentiation. Adipogenic differentiation was induced by treating H9‐MSCs seeded at 120,000 cells per cm^2^ with DMEM containing 10% FBS, 1 mM sodium pyruvate, 10^−6^ M dexamethasone, 10 μg/ml insulin, 0.5 mM isobutylmethylxanthine, 200 μM indomethacin, and 1% penicillin/streptomycin as previously described 43. At 21 days, adipogenic cultures were fixed and stained in Oil red O (Sigma‐Aldrich) for detection of lipid accumulation.

### High‐Density Chondrogenic Pellet Cultures

Chondrogenic differentiation was induced by culturing H9‐MSC as high‐density pellets (2.5 × 10^5^ cells per pellet) in DMEM‐HG (Thermo Fisher Scientific Life Sciences) supplemented with 1% ITS+ (ITS+ TM Premix, insulin‐transferrin‐selenium; BD Biosciences), 40 µg/ml L‐proline, 1 mM sodium pyruvate, 1% nonessential amino acids, 2 mM Glutamax, 50 µg/ml ascorbic acid 2‐phosphate, 10^−7^ M dexamethasone, and 1% penicillin/streptomycin as previously described 9, 26, 43 44 45. After 48 hours of pellet formation (day 0), cultures were left untreated in chondrogenic medium or treated with human recombinant BMP‐2 (100 ng/mL; ConnStem Inc., Cheshire, CT, http://www.connstem.com) or Wnt5a (50 ng/mL; R&D Systems, Minneapolis, MN, https://www.rndsystems.com). Medium was exchanged every other day. On days 0 (undifferentiated population), 5, 7, 10, and 14 of differentiation, pellets were harvested and fixed, and then embedded in paraffin and sectioned. Sections were stained with 1% Alcian Blue and Nuclear Fast Red solutions, or 0.1% Safranin O solution (Polysciences Inc., Warrington, PA, http://www.polysciences.com) for proteoglycan detection.

### Real‐Time Quantitative Polymerase Chain Reaction Analyses

H9‐MSC pellets at days 0, 5, 7, 10, and 14 were dissociated in TRIzol reagent (Thermo Fisher Scientific Life Sciences), and total RNA was isolated, treated with DNase I (Bio‐Rad, Hercules, CA, http://www.bio-rad.com), and reverse‐transcribed to cDNA by using iScript reverse transcriptase (Bio‐Rad) following manufacturer's protocols. Real‐time quantitative polymerase chain reaction (qPCR) was performed for cDNA samples using SYBR Green I (Roche, Indianapolis, IN, http://www.roche.com), gene‐specific primers ( supplemental online Table 1), and an Applied Biosystems 7500 fast cycler (Applied Biosystems, Foster City, CA, http://www.appliedbiosystems.com). Expression values were expressed as 2^ΔΔ ‐Ct^, with ΔΔ‐Ct defined as the difference in crossing threshold (Ct) values between each experimental sample and the untreated day 0 control sample using glyceraldehyde‐3‐phosphate dehydrogenase (GAPDH) as an internal reference standard. Values greater than 1.0 represent a fold‐change increase in gene expression, and values less than 1.0 represent a relative decrease in expression in comparison with the H9‐derived MSC progenitor day 0 cell pellets before growth factor treatment 43 44 45.

### Athymic Rat Chondral Defect Model

Animal use was conducted in accordance with federal and institutional guidelines. Chondral defects were generated in the medial femoral condyle in 90‐ to 100‐day‐old nude (athymic) rats (315–340 g weight) in accordance with protocol approval from the institutional animal care committee. Perioperative pain medication and antibiotics were administered in accordance with the approved animal protocol.

After provision of anesthesia (2% isoflurane), the left hind limb was shaved and prepared in a sterile fashion. A standard medial parapatellar incision was created, followed by lateral displacement of the patella for exposure. With the knee held in flexion, a 1.5‐mm biopsy punch was used to create a defect in the cartilage surface of the posterior, non‐weight‐bearing aspect of the medial femoral condyle. Care was taken to limit the depth of the defect to the cartilage lining with minimal intrusion of the subchondral bone. The operated animals were randomly assigned to one of three experimental groups (*n* = 5 per group): (a) empty defects; (b) defects filled with day 14 H9‐derived MSC pellets not exposed to growth factors (untreated pellets); and (c) H9‐derived MSC pellets pretreated for 2 days with BMP‐2, followed by 12 days of Wnt5a treatment. A single pellet was implanted into the chondral defect for each treatment group. Next, a fibrin sealant (Ethicon, Somerville, NJ, http://www.ethicon.com) was used to retain the transplanted pellets in the defect. The patella was reduced back into the trochlear groove, and the capsular and skin incisions were closed in separate layers. Rats were allowed weight bearing postoperatively and then euthanized at 4 or 8 weeks to harvest limbs for processing.

### Histological Assessments

Harvested limbs were cleaned and fixed in 10% formalin for 10 days. Intact joint specimens were decalcified for 2 weeks in 14% EDTA, then embedded in paraffin. Histologic sections, 7‐μm thick, spanning the entire joint were collected. Toluidine blue and Fast Green staining was performed to identify cartilaginous versus bony structures. Stained samples were imaged and analyzed to determine the extent of cartilage repair. Cartilage repair was graded blindly by two observers using an established grading scale 46. Histological scores were assigned a score from 0 to 14 based on five independent criteria (I: cell morphology [0–4]; II: metachromasia [0–3]; III: surface regularity [0–3]; IV: thickness of cartilage [0–2]; and V: integration with donor or host adjacent tissue [0–2]).

### Immunohistochemistry

We performed immunostaining to evaluate the composition of the extracellular matrix using the primary antibody anti‐collagen type II (EMD Millipore, catalog no. MAB8887). Enzymatic retrieval was performed by incubating the slides in pepsin solution (Thermo Fisher Scientific Life Sciences) at 37°C for 20 minutes. Slides were permeabilized with 0.1% Tween‐20 in PBS, blocked in 2.5% horse serum (Vector Laboratories, Burlingame, CA, http://vectorlabs.com; catalog no. S‐2012) for 1 hour, incubated with primary antibody (1:200) overnight at 4°C, and then incubated in horseradish peroxidase‐linked secondary antibody for 1 hour at room temperature; 1:200 Peroxidase anti‐mouse IgG1 (Vector Laboratories, catalog no. PI‐2000). Revelation was performed by using the 3‐amino‐9‐ethylcarbazol (RED) Substrate Kit (Thermo Fisher Scientific Life Sciences) following manufacturer's protocol. For detection of human cells within the rat chondral defect, we performed immunostaining using the human‐specific anti‐mitochondria antibody (EMD Millipore, catalog no. MAB1273). Enzymatic retrieval used sodium citrate (Sigma‐Aldrich) at a sub‐boiling temperature for 10 minutes, followed by incubation in a pepsin solution (Thermo Fisher Scientific Life Sciences) for 20 minutes at 37°C and immunostaining as described above.

### Gene Expression Analyses of the Reparative Tissue Harvested From Rat Chondral Defects

Articular cartilage from the chondral defects was scraped off histological slides at each time point. The harvested chondrocytes were lysed at 70°C in NP‐40 solution containing RNAsin plus (Promega) and pooled human mRNA‐specific PCR primers ( supplemental online Table 1). Lysates were treated with DNase I, and cDNA was prepared from total RNA by using Moloney murine leukemia virus reverse transcriptase (Thermo Fisher Scientific Life Sciences) following manufacturer's protocol. The samples were preamplified in multiplex for 16 cycles by using PCR master mix (TaKaRa Bio Inc., Shiga, Japan, http://www.takara-bio.co.jp) before single‐plex qPCR analysis 47, 48. GAPDH was used as an internal reference standard, and expression data graphed is relative to the parental H9‐MSC.

### Statistical Analysis

For gene expression analyses, we used student *t* tests or a one‐way analysis of variance, followed by a Bonferroni post hoc test, depending on whether there were two or three groups, respectively. Treatment groups were compared with untreated control samples at each time point. For histological scoring of cartilage repair in vivo 46, the nonparametric Mann‐Whitney *U* test was used to establish statistical significance of graded samples across independent observations of the repaired tissue made by two different observers. Data were expressed as mean ± SEM of three independent samples. Statistical significance was established at *p* ≤ .05.

## Results

### Derivation and Characterization of MSC‐Like Progenitor Cells From Human Embryonic Stem Cells

**Figure 1:**
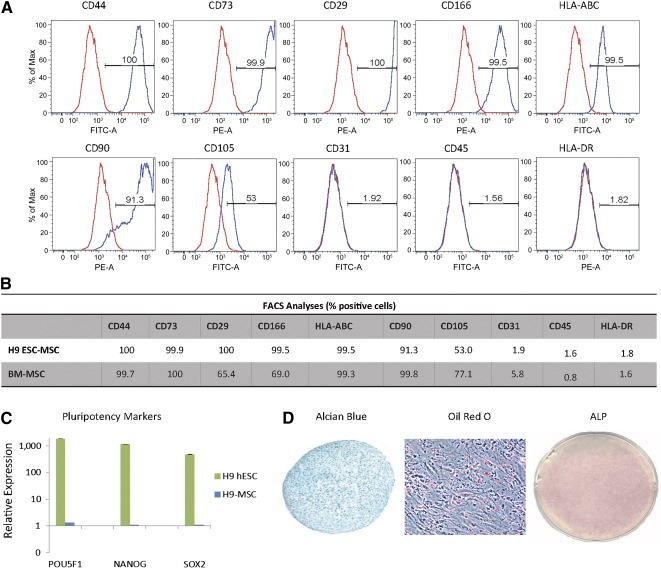
Mesenchymal stem cell‐like progenitors derived from H9 hESCs (H9‐derived MSCs) share common features with bone marrow‐derived MSCs. **(A):** Expression of cell surface antigens on progenitor cells by flow cytometry analysis. Histograms display percent cells expressing cell‐surface antigens of mesenchymal markers on H9‐derived MSC‐like cells (H9‐MSCs). H9‐MSCs express markers associated with the mesenchymal phenotype (CD44, CD73, CD29, CD166, CD90, CD105, and HLA‐ABC) and lack expression of endothelial (CD31) and hematopoietic markers (CD45 and HLA‐DR). Negative isotype controls are shown with percent fluorescence indicated for positive signal over isotype controls. **(B):** Comparative flow cytometry analyses of H9‐derived MSCs and the adult human bone marrow‐derived MSCs (Lonza) showed similar cell surface expression profiles. **(C):** Quantitative reverse‐transcriptase polymerase chain reaction analyses revealed downregulation of canonical pluripotency genes in H9‐derived MSC progenitors compared with undifferentiated H9 ESCs. **(D):** Alcian Blue staining of H9‐derived MSC high‐density pellets cultured in serum‐free chondrogenic medium for 21 days (magnification, ×4); Oil Red O staining of H9‐derived MSC progenitor monolayer cells cultured in adipogenic medium for 21 days (magnification, ×20); and a 10‐cm tissue culture dish (magnification, ×1) with alkaline phosphatase staining of H9‐derived MSC monolayer cells cultured in osteogenic medium for 21 days. Abbreviations: ALP, alkaline phosphatase; BM‐MSC, bone marrow‐derived mesenchymal stem cell; ESC, embryonic stem cell; FACS, fluorescence‐activated cell sorting; FITC‐A, fluorescein isothiocyanate A; hESC, human embryonic stem cell; HLA‐ABC, human leukocyte antigen ABC; Max, maximum; MSC, mesenchymal stem cell; PE‐A, phycoerythrin A.

Using the culture conditions we previously established for generating mesenchymal‐like stem cells from human induced pluripotent stem cells (iPSCs) 43 44 45, we generated an MSC‐like population from the human H9‐ESC line. H9‐MSCs were analyzed via flow cytometry to validate the expression of hallmark MSC cell‐surface antigens. H9‐MSCs displayed expression of cell surface markers characteristic of the adult mesenchymal stem cells, including CD44, CD73, CD29, CD166, and human leukocyte antigen ABC (HLA‐ABC) (Fig. 1A). H9‐MSCs uniformly expressed high levels (>90%) of all MSC markers, with the exception of CD105 (53% positive). Conversely, the H9‐MSCs exhibited minimal cell surface expression of endothelial marker (CD31) and hematopoietic lineage markers (CD45, HLA‐DR). These profiles were similar to that of adult human bone marrow‐derived mesenchymal stem cells (BM‐MSCs) (Fig. 1B). RT‐qPCR analyses revealed the suppression of pluripotency marker gene expression (*POU5F1*, *NANOG*, and *SOX2)* in H9‐MSCs relative to the undifferentiated parental H9 hESCs (Fig. 1C).

To demonstrate multipotency of the MSC‐like cells derived from H9 hESCs, we evaluated their ability to differentiate into chondrocytes, adipocytes, and osteoblasts in vitro. H9‐MSCs differentiated into chondrocytes, as evidenced by Alcian Blue staining of proteoglycan deposition in histological sections of high‐density pellet cultures cultured under chondrogenesis‐promoting conditions for 21 days. Similarly, H9‐derived MSC cultures exhibited Oil Red O staining of lipid droplets and alkaline phosphatase staining when cultured under adipogenic conditions and osteogenic conditions, respectively (Fig. 1D). In control samples, the undifferentiated H9‐MSCs did not exhibit histological staining indicative of the chondrogenic, osteogenic, or adipogenic lineages (data not shown).

### Controlled Commitment of H9‐Derived MSC‐Like Progenitors to the Chondrogenic Lineage by Wnt5a

**Figure 2:**
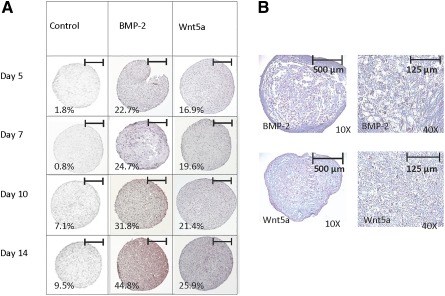
Wnt5a promoted chondrogenesis and limited hypertrophy in H9‐derived mesenchymal stem cell (MSC) pellets. **(A):** Analysis of H9‐MSC progenitors cultured as high‐density pellets for up to 14 days in serum‐free chondrogenic medium with or without BMP‐2 (100 ng/mL) or Wnt5a (50 ng/mL). Safranin O staining of 5‐μm histological sections of growth factor‐treated pellets at days 5, 7, 10, and 14 showed proteoglycan deposition in both the BMP‐2‐ and the Wnt5a‐treated samples. Quantification of percent area stained with Safranin O (*n* = 6) is shown for each time point recorded. Scale bars = 500 μm. **(B):** Alcian Blue staining with nuclear fast red counterstain showed presence of hypertrophic chondrocytes in BMP‐2‐treated pellets, which were not detected in the Wnt5a‐treated pellets.

To examine Wnt5a's ability to promote chondrogenic differentiation of human ESC‐derived progenitors, H9‐MSCs were cultured in high‐density pellets and treated with either Wnt5a or BMP‐2 (Fig. 2). Safranin O staining of histological sections revealed increased proteoglycan deposition in cultures treated with either Wnt5a or BMP‐2, compared with the untreated controls (Fig. 2A). After 14 days in pellet culture, the extent of proteoglycan staining was significantly higher in BMP‐2‐treated pellets (44.8% of total pellet area) compared with the control pellets (9.5% of total pellet area). However, the BMP‐2‐treated cultures also contained hypertrophic chondrocytes. In pellets treated with Wnt5a, 25.9% of the total pellet area revealed Safranin O staining, with little to no histological evidence of chondrocyte hypertrophy (Fig. 2B).

**Figure 3:**
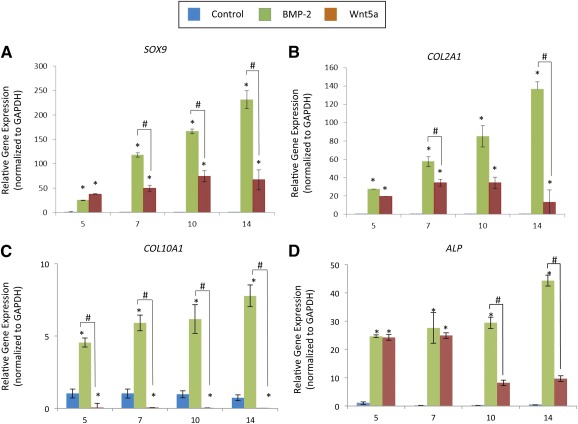
Wnt5a induced chondrogenic gene expression and limited expression of the hypertrophic chondrocyte markers in H9‐derived MSC pellets. Comparative expression of early chondro‐progenitor genes *SOX9* **(A)** and *COL2A1* **(B)** and the expression of mature chondrocyte markers of hypertrophy *COL10A1* **(C)** and *ALP* **(D)** in H9‐derived MSC pellets cultured for 14 days with or without 100 ng/ml BMP‐2 or 50 ng/ml Wnt5a. Values greater than 1.0 represent a fold‐change increase in gene expression and less than a 1.0 relative decrease in expression in comparison with the undifferentiated H9‐derived MSCs (day 0). ∗, *p* < .05 (treatment compared with untreated controls); #, *p* < .05 (statistical significance between treatment groups). Abbreviations: ALP, alkaline phosphatase; GAPDH, glyceraldehyde‐3‐phosphate dehydrogenase.

Gene expression analyses of markers of early chondrogenesis revealed that treatment with either Wnt5a or BMP‐2 significantly induced *SOX9* and *COL2A1* expressions at all time points, compared with untreated controls (Fig. 3A, 3B). This induction was more pronounced for BMP‐2‐treated pellets, compared with Wnt5a‐treated pellets. Consistent with our histological evaluations, the hallmark marker of chondrocyte hypertrophy (*COL10A1)* was up‐regulated by approximately eightfold in the day 14 BMP‐2‐treated pellets, but this was not seen in Wnt5a‐treated pellets (Fig. 3C). *ALP* expression, indicative of matrix maturation, was also significantly higher in day 14 BMP‐2‐treated pellets (44‐fold induction compared with control), as compared with Wnt5a‐treated pellets (ninefold induction compared with control) (Fig. 3D).

**Figure 4:**
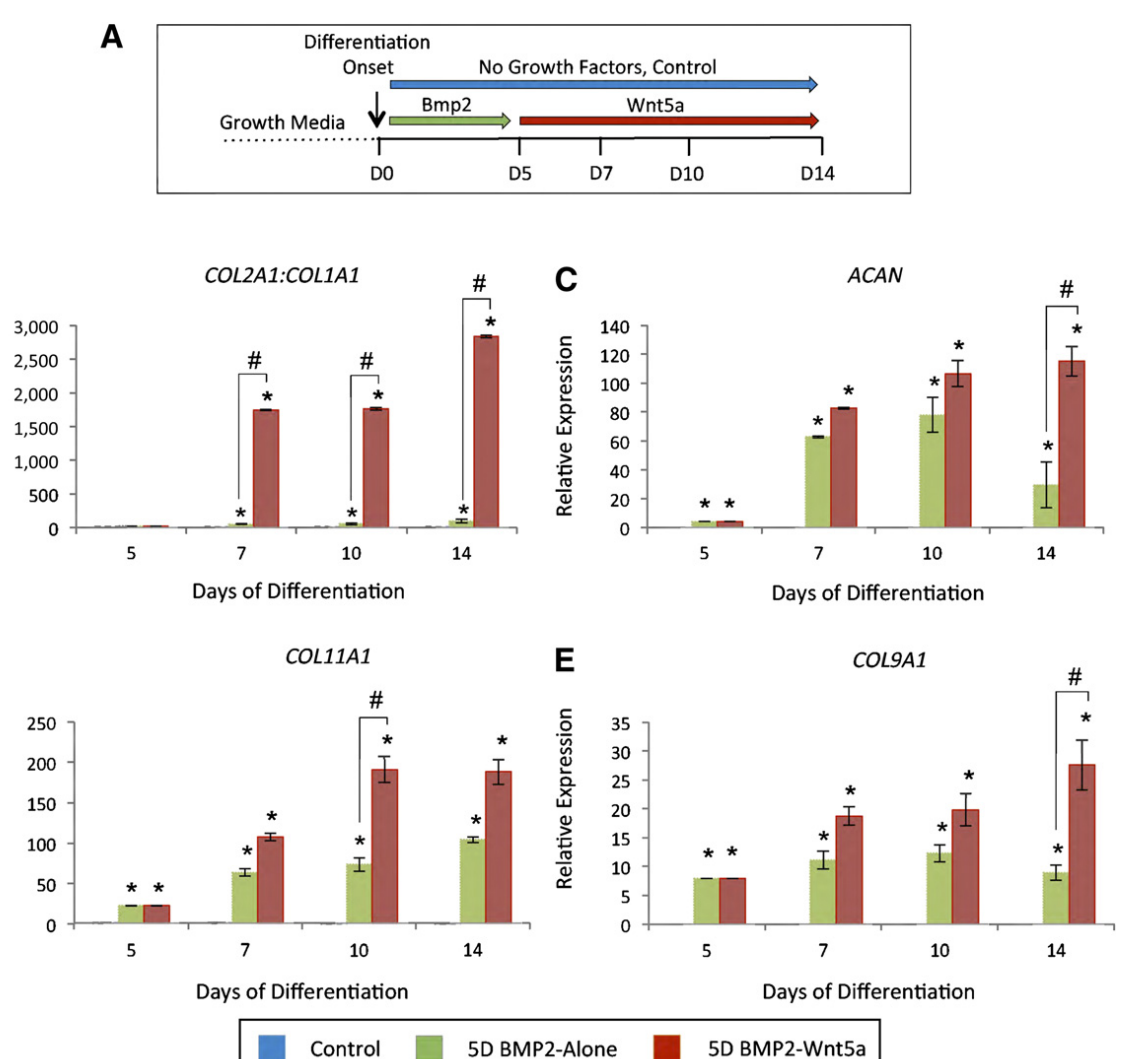
Sequential treatment with BMP‐2 followed by Wnt5a induces sustained expression of chondrocyte matrix markers under chondrogenic conditions. **(A):** Schematic shows treatment conditions for in vitro chondrogenic differentiation of H9‐MSCs. **(B–E):** Expression data based on quantitative polymerase chain reaction analyses of cartilage matrix genes in treatment groups from differentiating H9‐derived mesenchymal stem cell (MSC) pellets cultured for 5 days in 100 ng/ml BMP‐2 followed by up to 9 days of no growth factor treatment (5D BMP2‐Alone) or sequential treatment with 50 ng/ml Wnt5a (5D BMP2‐Wnt5a): ratio of *COL2A1:COL1A1* **(B)**, *ACAN* **(C)**, *COL11A1* **(D)**, and expression of *COL9A1* **(E)**. Values greater than 1.0 represent a fold‐change increase in gene expression, and those less than 1.0 indicate a relative decrease in expression in comparison with the undifferentiated H9‐derived MSCs (day 0). ∗, *p* < .05 (treatment compared with untreated controls); #, *p* < .05 (statistical significance between treatment groups). Abbreviation: D, day.

**Figure 5:**
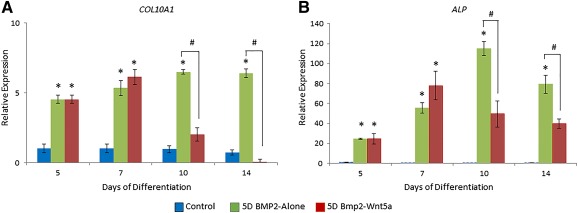
Wnt5a suppressed BMP‐2‐induced markers of chondrocyte hypertrophy. Comparative expression of mature chondrocyte markers of hypertrophy *COL10A1* **(A)** and *ALP* **(B)** in H9‐derived mesenchymal stem cells (MSCs) cultured for 5 days in 100 ng/ml BMP‐2, followed by up to 9 days of no growth factor treatment (5D BMP2‐Alone) or sequential treatment with 50 ng/ml Wnt5a (5D BMP2‐Wnt5a). Values greater than 1.0 represent a fold‐change increase in gene expression and less than 1.0 indicate a relative decrease in expression in comparison with the undifferentiated H9‐derived MSCs (day 0). ∗, *p* < .05 (treatment compared with untreated controls); #, *p* < .05 (statistical significance between treatment groups). Abbreviations: ALP, alkaline phosphatase; 5D, 5 days.

After individual treatment with BMP‐2 and Wnt5a, H9‐MSC pellets were treated sequentially with BMP‐2 for 5 days, followed by Wnt5a for 9 days (Fig. 4A). This was done to determine whether sequential treatment could induce the expression of genes associated with an articular chondrocyte phenotype rather than growth plate‐like chondrocytes. The *COL2A1*:*COL1A1* ratio was significantly higher after sequential treatment at each time point (Fig. 4B). Similarly, *COL11A1*, *COL9A1*, and *ACAN* expression were also significantly enhanced (threefold for *COL11A1* at day 10, threefold for *COL9A1* at day 14, and fourfold for *ACAN* at day 14) after sequential treatment, compared with BMP‐2 alone (Fig. 4C–4E). In contrast, the sequential treatment with BMP‐2 followed by Wnt5a resulted in threefold and 128‐fold reduced expression of *COL10A1* at 10 and 14 days, respectively, compared with treatment with BMP‐2 alone (Fig. 5A, 5B). Similarly, sequential treatment resulted in a twofold reduction of *ALP* expression at day 14, compared with BMP‐2 alone.

### Cartilage Repair Generated by Pretreated H9‐Derived MSC Pellets in a Rat Chondral Defect Model

**Figure 6:**
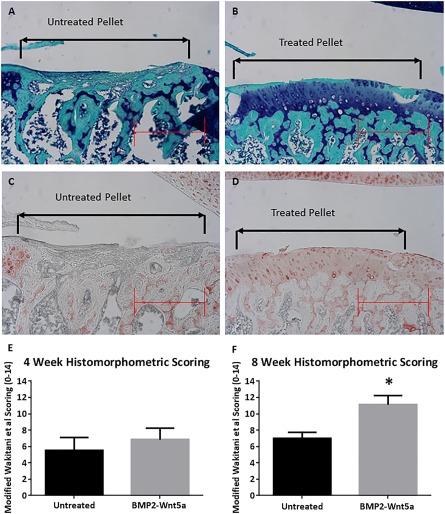
Representative gross morphometric images (magnification, ×10) and collagen type II immunostaining demonstrate enhanced cartilage repair mediated by H9‐derived mesenchymal stem cell (MSC) pellets pretreated with sequential BMP‐2 and Wnt5a in the rat chondral defect model at 8 weeks. Scale bars = 500 μm. **(A, B):** Toluidine blue and Fast Green staining of untreated **(A)** and pretreated (2 days of BMP‐2 followed by 12 days of Wnt5a) implanted H9‐derived MSC pellets **(B)** at the defect site showed enhanced staining for the treatment group. **(C, D):** Collagen type II immunohistochemistry of the untreated **(C)** and sequentially treated pellets **(D)** demonstrates significant staining for the treatment group and the absence of staining for the untreated control. **(E, F):** Mean histological scores obtained using a published grading scale for cartilage repair at 4 week **(E)** and 8 weeks **(F)** 46. ∗, Total histological scores of chondral defect repair exhibited significance of chondral repair in the pretreatment group over the untreated group at 8 weeks.

To examine whether our in vitro strategy could promote H9‐derived MSC‐mediated repair of articular cartilage in vivo, we used our pellet culture treatment strategy to treat femoral chondral defects created in an athymic nude rat model ( supplemental online Fig. 1). Histological staining with Toluidine blue and immunohistochemical staining with anti‐collagen type II antibodies were used to assess tissue composition of the reparative tissue. Our histological analyses revealed that chondral defects filled with untreated H9‐derived MSC‐implanted pellets led to formation of fibrous‐like tissue with flattened cells, a disorganized superficial zone, and negligible collagen type II staining of the extracellular matrix at 8 weeks after surgery (Fig. 6A, 6C). In contrast, the defects filled with sequentially treated H9‐derived MSC pellets exhibited the histological appearance of typical rounded chondrocytes, a smooth superficial zone appearance, and prominent collagen type II staining within the extracellular matrix (Fig. 6B, 6D). Using a well‐established grading scale 46, we evaluated the degree of articular cartilage repair among the animal groups. Histological grading of the defects implanted with H9‐MSC pellets treated with BMP‐2 followed by Wnt5a showed significantly higher scores than the untreated H9‐MSC pellet controls at 8 weeks (Fig. 6F). No significant difference in histological grading was observed between untreated H9‐MSC pellet controls and the treatment group at 4 weeks postsurgery (Fig. 6E, supplemental online Fig. 3).

**Figure 7:**
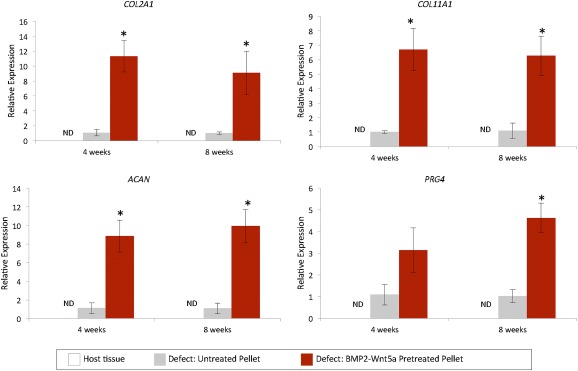
Regeneration of permanent cartilage‐like tissue by implanted H9‐derived mesenchymal stem cells (MSCs) pretreated with sequential BMP‐2 and Wnt5a. Quantitative reverse‐transcriptase polymerase chain reaction of preamplified tissue scraped from the defected regions on prepared slides of paraffin‐embedded rat knees 4 and 8 weeks after surgery is shown. Human‐specific expressions of the early chondrogenic gene marker *COL2A1* and articular chondrocyte matrix markers *COL11A1*, *ACAN*, and *PRG4* were detected in only in tissue scrapings from animals receiving the untreated human H9‐derived MSCs, and the human H9‐derived MSCs pretreated for 14 days (2 days, BMP‐2, 12 days, Wnt5a). Values greater than 1.0 represent a fold‐change increase in gene expression and less than 1.0 indicate a relative decrease in expression in comparison with the undifferentiated H9‐derived MSCs (day 0). ∗, *p* < .05. Abbreviation: ND, no detection of genes in host rat cartilage tissue using human‐specific gene primers.

Finally, to confirm the engraftment of the implanted H9‐derived chondrocytes, defects were stained with a human‐specific mitochondrial antibody. No signal was detected in host tissue, whereas staining was detected in the repair tissue in animals implanted with H9‐MSC pellets ( supplemental online Fig. 2). The human origin of the repair tissue formed within area of the defect was also verified via quantitative RT‐PCR analyses using species‐specific gene primers (Fig. 7). Tissue recovered from the defect sites implanted with H9‐MSC pellets treated with BMP‐2 followed by Wnt5a displayed increased expression of human specific *COL2A1*, *ACAN,* and *COL11A1* as compared with untreated pellet implants at 4 and 8 weeks. Furthermore, at 8 weeks after surgery, animal groups receiving implants sequentially treated with BMP‐2 and Wnt5a demonstrated increased levels of *PRG4*. The expression of these human genes was not detected in host tissue used as a negative control.

## Discussion

Although several studies have evaluated the efficacy of stem cell treatment, with and without the use of scaffolds, to regenerate articular cartilage in osteochondral defects 28 29 30 31, no studies have demonstrated the unequivocal formation of articular‐like cartilage in the absence of chondrocyte hypertrophy. In this study, we developed an effective strategy to promote the generation of articular‐like cartilage based on the sequential treatment of differentiating mesenchymal progenitors from hESCs with BMP‐2 and Wnt5a.

Currently, autologous chondrocyte implantation and matrix‐induced autologous chondrocyte implantation (MACI) are the only cell‐based repair strategies for articular cartilage regeneration in clinical practice. The long‐term results of MACI are currently unknown, given that it is a new technology. ACI, however, has not consistently regenerated hyaline articular cartilage. At a second‐look biopsy 2 years after undergoing ACI, only 6 of 32 (18.8%) patients demonstrated “mostly hyaline cartilage,” whereas 16 of 32 (50%) patients demonstrated fibrocartilage, no repair tissue, or bone 49. Given these results, clinicians have increasingly relied upon procedures such as osteochondral autograft and allografting to repair cartilaginous lesions. The requisite expansion of biopsied chondrocytes in vitro for ACI has been associated with dedifferentiation, differential gene expression, and loss of the cell surface markers typically displayed in articular chondrocytes 50 51 52 53 54. Uncontrolled redifferentiation of these expanded chondrocytes may cause these suboptimal histologic outcomes.

A body of evidence is available regarding the ability of somatic stem cells to yield articular‐like chondrocytes. However, a major limiting factor to using adult mesenchymal stem cells for cartilage repair in the clinic has been the variable capacity of somatic cells to regenerate permanent cartilage 43, 55 56 57 58 59. To this end, various groups have explored the use of pluripotent stem cells for cartilage repair 20, 23, 24, 28 29 30 31, 60 61 62. Specifically, multiple methods have been proposed to derive chondrocytes from human embryonic and induced pluripotent stem cells 45, 63. However, the level of success has relied on the successful derivation of multipotent mesenchymal progenitors from these pluripotent cells. We used a direct plating method to generate mesenchymal‐like stem cells from the H9 embryonic stem cell line 43 44 45. We selected the H9 cell line to understand multipotent directed differentiation from a developmental perspective. H9‐derived MSC‐like cells showed loss of expression of the pluripotency genes, while exhibiting features of adult MSCs, including their ability to give rise to mesenchymal derivatives such as bone, fat, and cartilage 45, 60, 63. In our hands, these mesenchymal progenitors were responsive to chondrogenic stimuli, as previously shown with human mesenchymal stem cells 10 11 12, 14, 36, 64.

Singular treatments of the H9‐MSC pellets with either BMP‐2 or Wnt5a were less effective in pushing the cells toward an articular‐like chondrocyte phenotype as compared with their sequential treatment. Exposure to human recombinant BMP‐2 induced a robust chondrogenic response; however, sustained exposure to the BMP‐2 resulted in the induction of chondrocyte maturation. This is in agreement with previous reports showing that BMP‐2 enhances the expression of cartilage‐specific genes in various sources of multipotent stem cells. However, the ability of BMP‐2 to mediate hyaline cartilage matrix production has not been established 22, 32, 43, 60, 64 65 66 67. Treatments with Wnt5a alone elicited a less potent chondrogenic response compared with BMP‐2. However, exposure of the cell pellets to Wnt5a did not promote chondrocyte hypertrophy in vitro. We and others have previously reported chondrogenic effects of Wnt5a on mesenchymal limb condensations, through the noncanonical pathway. In this model, we further demonstrated Wnt5a‐mediated inhibition of chondrocyte hypertrophy through stage‐specific inhibition of *Runx2* by nuclear factor‐κB 38 39 40. To this effect, we postulated that Wnt5a may inhibit BMP‐2 induction of terminal maturation. In our human stem cell model, Wnt5a antagonized BMP‐2‐mediated induction of chondrocyte hypertrophy in vitro. Based on this model, we established conditions in which transient exposure to BMP‐2 was necessary to initiate chondrogenic differentiation and early matrix formation, while Wnt5a prevented terminal maturation of these cells. Although the expression of markers of hyaline cartilage matrix such as collagen types IX and XI in these pellets in vitro was unexpected, it was highly encouraging to establish an in vivo correlate to our in vitro findings.

Significant advances in the understanding of the directed differentiation of human ESCs and iPSCs to chondrocytes have been made. However, the regeneration of permanent cartilage has not yet been ascertained 11, 20, 23, 24, 63. Recently, Craft et al. 68 described the generation of articular‐like chondrocytes from iPSCs in vitro. Using embryoid bodies treated with Activin, BMP‐4, and bFGF, followed by treatment of monolayer cells with BMP‐4, bFGF, and hedgehog inhibitors, they successfully derived MSC‐like populations. Although these cells were not evaluated in a translational model of cartilage repair, the authors demonstrated features of hyaline cartilage with subcutaneous implantation of micromass cells treated with BMP‐4 and TGF‐β. Likewise, Yamashita et al. 69 demonstrated the derivation of iPSCs to chondroprogenitors through consecutive treatment with BMP‐2, TGF‐β, and GDF5 in high density. When these cells were surgically implanted into articular cartilage defects in immunosuppressed mini‐pigs, the authors noted an articular‐like cartilage phenotype. Although these results are encouraging, the authors stated that biointegration into the host tissue was not demonstrated. However, when another group utilized this same derivation method to obtain chondrocytes from human iPSCs, they noted teratoma formation after implantation of pellets treated with BMP‐4 followed by GDF5 in vivo 61.

Our in vivo data demonstrated that implantation of cell pellets treated with BMP‐2 followed by Wnt5a into defects created in the articular cartilage of nude rats resulted in the regeneration of cartilage with hyaline‐like features. This was supported by histologic scoring of the repair tissue, as well as molecular evidence of proteoglycan formation within the chondral defect and expression of human hallmark genes of hyaline cartilage in vivo. Future studies using a larger animal will ascertain whether this scaffoldless stem cell‐based therapy will yield mechanically stable structures and extend the duration of postimplantation evaluations to further demonstrate that the repair in larger animals can yield permanent, rather than transient, cartilage or fibrous tissue.

## Conclusion

The quest for establishing a clinically viable strategy for joint cartilage regeneration using various sources of progenitors continues with significant progress. Our sequential treatment of progenitor cells demonstrated a hyaline‐like articular cartilage phenotype in vivo. This specific treatment method as well as other strategies will likely evolve toward limiting the number of growth factors used to control cell differentiation and their potential replacement with small molecules that can be combined with bioengineered matrices to improve tissue integration and prolong cartilage integrity.

## Author Contributions

J.D.G., M.B.O., and F.A.: conception and design, collection and/or assembly of data, data analysis and interpretation, manuscript writing, final approval of manuscript;

D.N.P. and R.Y.: collection and/or assembly of data, data analysis and interpretation, manuscript writing, final approval of manuscript; R.M.G.: conception and design, data analysis and interpretation, manuscript writing, final approval of manuscript; H.D.: conception and design, financial support, administrative support, data analysis and interpretation, manuscript writing, final approval of manuscript.

## Disclosure of Potential Conflicts of Interest

The authors indicated no potential conflicts of interest.

## Supporting information

Supporting Information
