# Supplementary material for: Regeneration of Articular Cartilage by Human ESC‐Derived Mesenchymal Progenitors Treated Sequentially with BMP‐2 and Wnt5a
Source: Stem Cells Transl Med. 2016 Aug 5;6(1):40–50. doi: 10.5966/sctm.2016-0020 (PMC5442752; doi:10.5966/sctm.2016-0020)
Supplement: Supplementary file 1 — Supporting Information [file SCT3-6-040-s001.pdf]

Supplemental Information – Drissi et al.

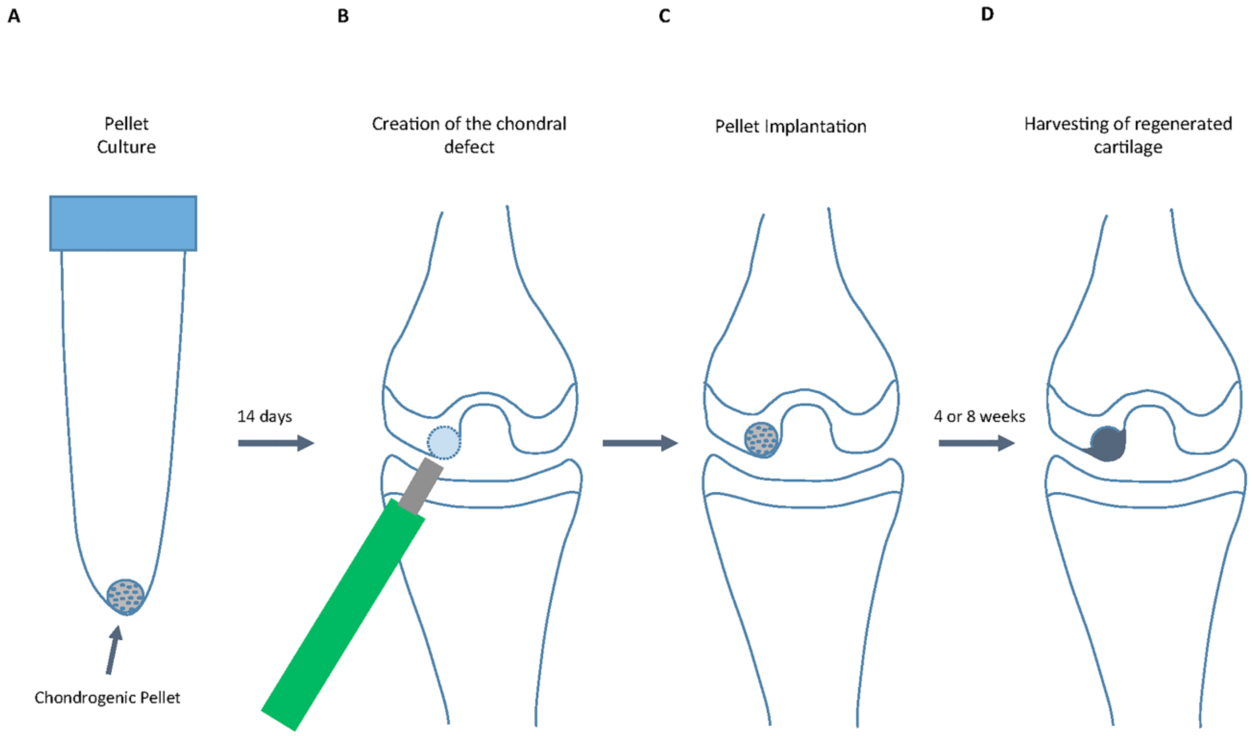

Figure S1: The nude rat in vivo chondral defect model is depicted. H9-derived MSC pellets were cultured in chondrogenic media for 14 days, with or without sequential treatment of 2 days of BMP-2 followed by 12 days of Wnt5a (A). NIH-RNU nude rats 12 weeks of age were anesthetized, a medial parapatellar incision was made, and the patella was dislocated laterally to expose the articular surface. A 1.5mm unilateral defect was made in the medial femoral condyle using a biopsy punch (B), and the H9-derived MSC pellets were implanted into the defect (2 pellets per animal) using a fibrin sealant, and the capsular and skin incisions were closed (C). The animals were sacrificed at 4 and 8 weeks for histologic evaluation (D).

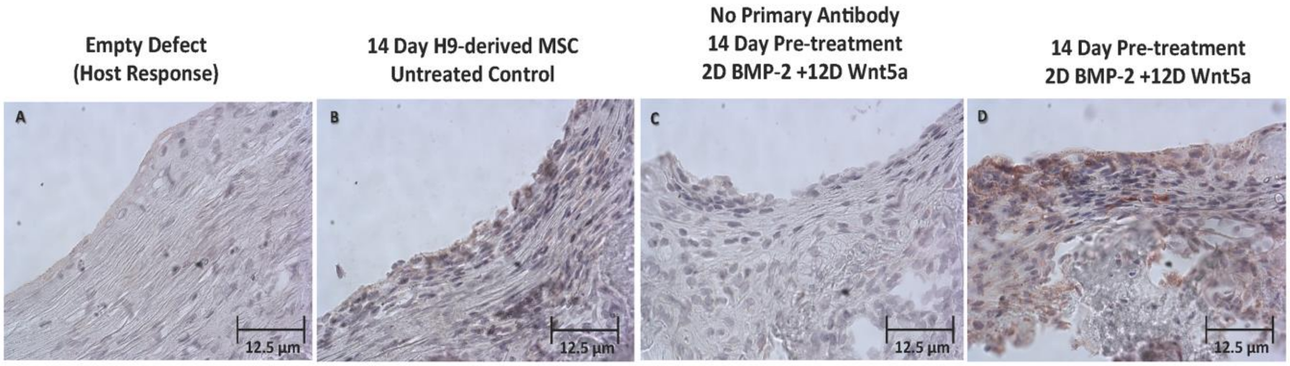

Figure S2: Representative gross morphometric images (40X) of rat chondral defects at 4 weeks post-surgery. Human mitochondrial-specific antigen staining of prepared slides for tissue within the defected regions from animals serving as a defect control (A), and animals receiving either the untreated control H9-derived MSC implant (B), or the BMP-2 and Wnt5a sequentially treated H9-derived MSC implant without primary antibody (C), and with primary antibody (D). Regenerated tissue contributed by the H9-derived MSC implants is evidenced by the staining of the perinuclear regions of cells within the defect.

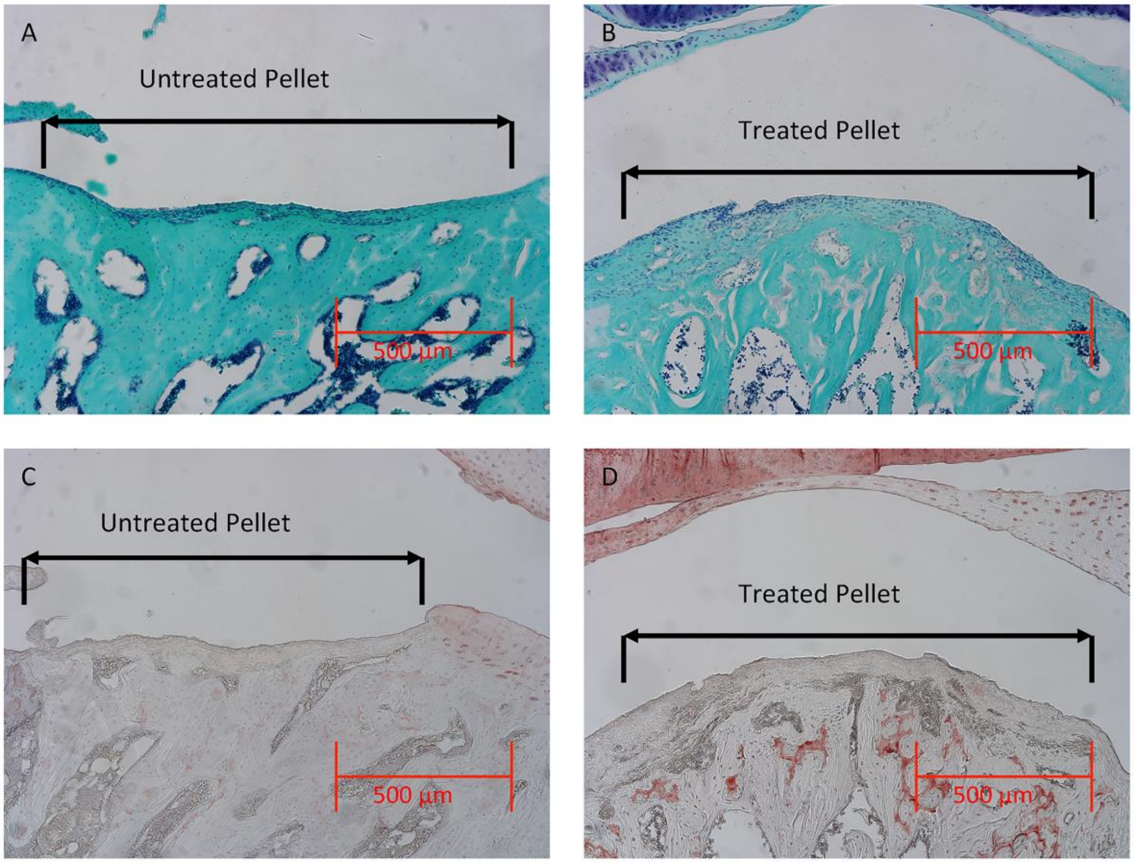

Figure S3: Representative gross morphometric images (10X) with Toluidine Blue and Fast Green staining of rat chondral defects at 4 weeks post surgery for the untreated (A) and pretreated (2 days of BMP-2 followed by 12 days of Wnt5a) implanted H9-derived MSC pellets (B). There is increased cellularity at the defect site for the treatment group. However, there is limited production of extracellular matrix. Collagen type II immunohistochemistry of the untreated (C) and sequentially treated pellets (D) demonstrates an absence of staining at the defect site at 4 weeks.

Supplemental Table

Table S1: List of quantitative PCR primer pairs and corresponding gene accessions used for gene-specific RT-PCR analyses.

Gene (accession no.)	Forward Primer	Reverse Primer
GAPDH **(NM_002046)**	aattccatggcaccgtcaag	agggatctcgctcctggaag
POU5F1 **(NM_203289)**	tgtactcctcggtccctttc	tccaggttttctttccctagc
NANOG **(NM_024865)**	cagtctggacactggctgaa	ctcgctgattaggctccaac
ALP **(NM_001177520)**	gacaagaagcccttcactgc	agactgcgcctggtagttgt
SOX9 **(NM_000346)**	agacagcccccctatcgactt	cggcaggtactggtcaaact
ACAN **(NM_013227)**	tcgaggacagcgaggcc	tcgagggtgtagcgtgtagaga
COL2A1 **(NM_001844)**	ggcaatagcaggttcacgtaca	cgataacagtcttgccccactt
COL11A1 **(NM_001854)**	ccccagagccagcccactgag	cctcttcacctggggtggggt
COL9A1 **(NM_001851)**	ttttagccctcaccgggggca	agtcactgtcccctcacgaccc
COL1A1 **(NM_000088)**	gtgctaaaggtgccaatggt	accaggttcaccgctgttac
COL10A1 **(NM_000493)**	caaggcaccatctccaggaa	aaagggtatttgtggcagcatatt
